# Effect of Peak Current Density on Tensile Properties of AZ31B Magnesium Alloy

**DOI:** 10.3390/ma14061457

**Published:** 2021-03-17

**Authors:** Ichsan Indhiarto, Tetsuhide Shimizu, Ming Yang

**Affiliations:** Department of Mechanical System Engineering, System Design, Tokyo Metropolitan University, Tokyo 191-0052, Japan; indhiarto-ichsan@ed.tmu.ac.jp (I.I.); simizu-tetuhide@tmu.ac.jp (T.S.)

**Keywords:** magnesium alloy, electroplasticity, uniaxial tensile test, peak current density

## Abstract

An investigation into the effects, including the athermal effect, of a pulsed current on AZ31B magnesium alloy was carried out. Different peak current densities were applied at the same temperature under uniaxial tensile testing. The results indicate that the stress reduction caused by the increasing peak current density is independent of temperature. The strain hardening coefficient also shows a similar trend. The fracture strain shows the optimum value due to the current crowding effect.

## 1. Introduction

Due to its biocompatibility and biodegradable properties [1], there has been a surge of interest in incorporating AZ31B magnesium alloy into various forming processes. Despite this, its application is still limited by its poor plasticity, induced by its hexagonal close-packed (hcp) structure. The most common method of improving its plasticity is to form it at elevated temperatures. This gives rise to other issues, such as low accuracy, low oxidation, and long forming time. Resistance heating was applied to minimize the heating time, especially in the sheet metal formation process. This opens up a new discussion on the effects caused by current flowing into metals.

The relationship between dislocation movement and the flow of electricity in metals was observed in the 1960s [2]. It was also suggested that the interaction between electrons and the elastic field of the dislocation can promote the movement of some of the dislocation that had been hindered [3]. This effect of the current can be referred to as electroplasticity [4]. It was observed that electricity can improve the forgeability of various metals, such as aluminum, copper, iron, titanium-based alloys [5], and magnesium alloys [6]. Further investigation also showed that an increase in current density can reduce the springback angle in ultra-high-strength steels [7], commercially pure titanium [8], Ti-6Al-4V titanium alloy [9], and AZ31B magnesium alloy [10]. In contrast, a reduction in flow stress was also observed in various metals [11,12,13,14]. Despite widespread acceptance of the electroplasticity effect and its role in improving the formability of metals, there is still no satisfying evidence to distinguish between the effect of Joule heating and the effect of electroplasticity itself.

The objective of this study is to investigate the effects of a pulsed current on AZ31B magnesium alloy, including, additionally, the pulsed current’s athermal effect. A uniaxial tensile test was carried out using several peak current densities while maintaining the temperature. In order to determine the dependence on temperature, we also sought to vary the global temperature. The alloy’s ultimate tensile strength, strain hardening exponent, and fracture strain were analyzed.

## 2. Experimental Methods

**Figure 1:**
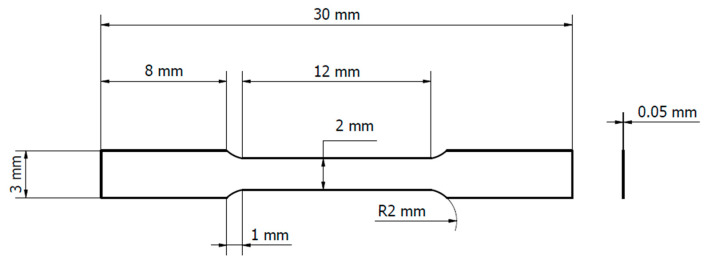
Specimen shape and dimensions.

The AZ31B used in this study was purchased from the Nippon Kinzoku Co., Ltd. (Itabashi Plant, Tokyo, Japan). Its chemical composition can be seen in Table 1. The tensile specimen was manufactured according to a DIN 5012 type H tensile test standard, as can be seen in Figure 1. The sheet of 50 µm thickness was cut in its rolling direction. The specimen was heat-treated for 1 h at 200 °C, then cooled to room temperature, both in vacuum. The backside was spray-painted with an emissivity of 0.94.

**Figure 2:**
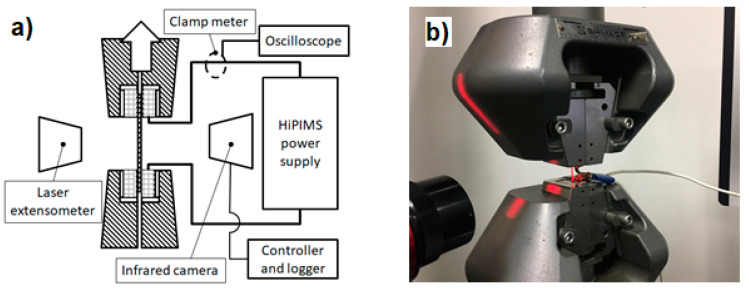
(**a**) Schematic and (**b**) real view of experimental conditions.

A schematic and real view of the experimental setup can be seen in Figure 2a,b, respectively. Uniaxial tensile testing was conducted using a universal test machine from Zwick Roell Z005, equipped with a Zwick Roel LaserXtens from ZwickRoell GmBH, Fürstenfeld, Austria as the extensometer and a Zwick Roell 5 kN load cell. The strain rate was 0.001 1/s for every experiment. An infrared thermography camera from Optris GmBH, Berlin, Germany was used to measure and record the surface temperatures of the specimens. The temperature reading was fed into the OMRON digital controller in Proportional-Integral-Derivative (PID) mode. This temperature control system was responsible for maintaining the temperature of the specimens. The pulsed current was provided by the PEKURIS PVP 2KV1KA pulse power supply. The positive pole was attached to the bottom side of the clamp.

**Figure 3:**
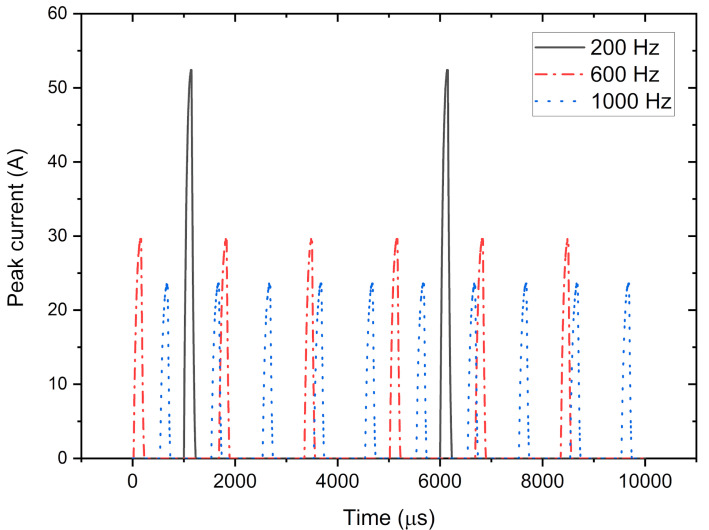
Schematic illustration of the pulsed pattern applied in this study.

A uniaxial test was conducted at different temperatures to assess the temperature dependency of the pulsed current effect. At each temperature, three different frequencies were applied. Several frequencies were used to achieve different peak current densities while maintaining a similar maximum global temperature using a PID controller. Using this setup, the effect of the pulsed current can be investigated separately from the Joule heating effect. Details of experimental parameters can be seen in Table 2. The schematic of the pulsed pattern can be seen in Figure 3.

## 3. Results and Discussions

### 3.1. Thermal Behavior and Current Density

The global temperature was measured in the sample’s widest area along the gauge length. The maximum temperature was maintained from the introduction of tension until fracture using a PID controller. The recorded temperature showed no noticeable deviation from the target temperature in all parameters. This mean temperature was successfully controlled to not exceed the target temperature throughout the experiment. The influence of global Joule heating in giving rise to different peak current densities at the same temperature can be safely ignored.

**Figure 4:**
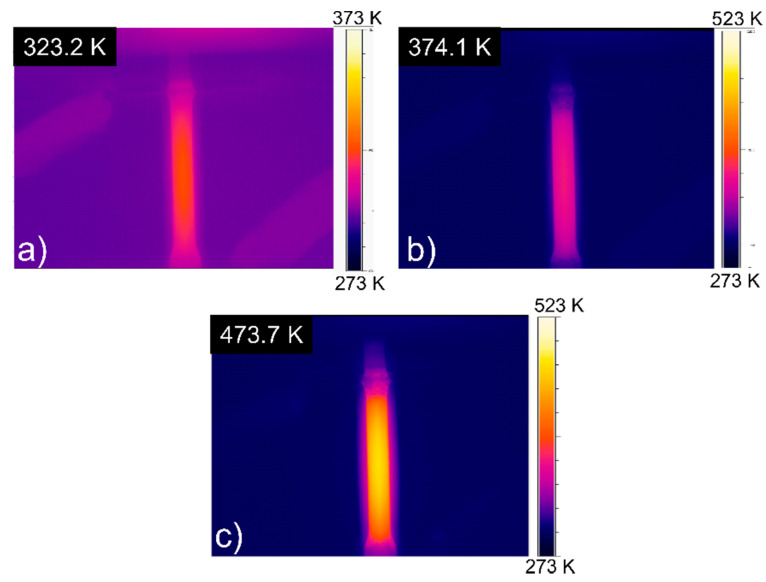
Representative picture of thermal imaging with recorded maximum values prior tension for (**a**) 323 K, (**b**) 373 K, and (**c**) 473 K.

**Figure 5:**
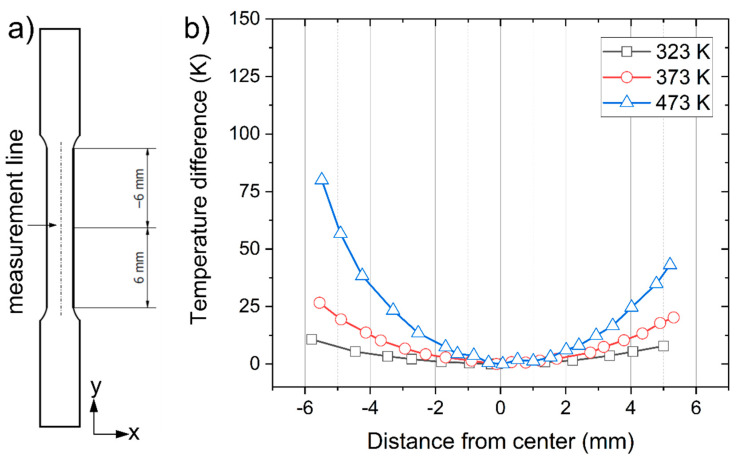
(**a**) Measurement line of thermal distribution, and (**b**) thermal distribution along the measurement line.

The temperature distribution is shown in Figure 4. The temperature was shown to not be evenly distributed and tended to decrease as we move further from the center. Figure 5a shows the position of the measurement line, and Figure 5b shows the temperature distribution along that line. Temperature difference for 323 K, 373 K, and 473 K was 10.7 K, 26.6 K, and 80 K, respectively. This phenomenon occurred because the heat was escaping in the direction of each upper and bottom jig. However, since the region of interest is in the middle of the specimens, where necking usually starts, this temperature distribution was ignored in our further analysis.

The heat generation caused by Joule heating can be approximated using the formula from [13]:

(1) $$T=J^{2}\frac{\rho_{e}d}{2h}+T_{\infty }$$

where T is temperature, $\rho_{e}$ is electrical resistivity, d is the specimen’s thickness, h is the heat convective coefficient, $T_{\infty }$ is the ambient temperature, and J is the current density, which can be expressed by

(2) J=I/A.

where I and A are the current and the specimen’s cross-sectional area, respectively.

It is noteworthy that $T\propto J^{2}$. This relationship pertains to an environment of direct current (DC) where there is no frequency domain. If a frequency domain exists, the peak value needs to be changed into its root mean square (RMS). The RMS value of the current density element can be calculated using

(3) $$J_{RMS}=J_{peak}\sqrt{D}$$

where D as in its duty cycle which is a ratio between the pulse width and the period. Thus, Equation (1) can be rewritten as,

(4) $$T=J_{RMS}^{2}\frac{\rho_{e}d}{2h}+T_{\infty }.$$

According to the experimental methods, the parameters were controlled by the target temperature and frequency. However, using the peak current density and its RMS counterpart enabled a better analysis of the effect of the pulsed current. The results in this study will be analyzed using the sample’s peak current density value rather than its frequency.

**Figure 6:**
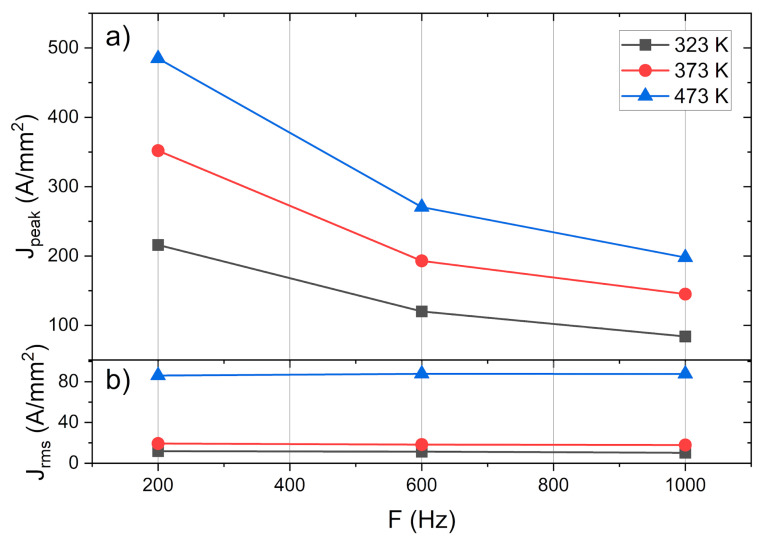
(**a**) Peak current density measured prior to tension and (**b**) its root mean square value.

The peak current density and RMS value can be seen in Figure 6a,b, respectively. The peak current density decreases as the frequency increases, and there is no noticeable change in RMS value even when the frequency is significantly changed. It can be concluded that the global temperature was strictly related to the RMS value, rather than the peak value.

Global temperature is different from local temperature, which occurs on a microscale. Local temperature is generated in the specimen’s interior at a given time, when electrons collide with defects in the lattice, resulting in the scattering of electrons. The heat generated by this scattering of electrons propagates via thermal vibration, resulting in heat distribution. The global temperature is the apparent temperature that is measured on a specimen’s surface. Local heat generation has been confirmed in the past by grain boundary melting [15]. In this study, we focus on the global heat generation aspect of Joule heating. That being said, the heat effect of Joule heating is neglected in this analysis.

The skin effect occurs when a pulsed current flows through a conductor. Its value can be determined via

(5) $$\delta =\sqrt{\frac{2\rho }{\omega \mu }}\;\sqrt{\sqrt{1+{\left(\rho \omega \epsilon \right)}^{2}}+\rho \omega \epsilon }.$$

where δ is the skin depth whereat the current density is 1/e of the value at the surface, ρ is the resistivity of the conductor, ω is the angular frequency of the current, μ is the permeability of the conductor, and ϵ is the permittivity of the conductor. The calculated skin depths for 200 Hz, 600 Hz, and 1000 Hz are 8.2 mm, 4.7 mm, and 3.67 mm, respectively. The thickness and width of the specimen are 50 µm and 2 mm, respectively, meaning a skin effect was not observed in this study.

### 3.2. Tensile Properties

**Figure 7:**
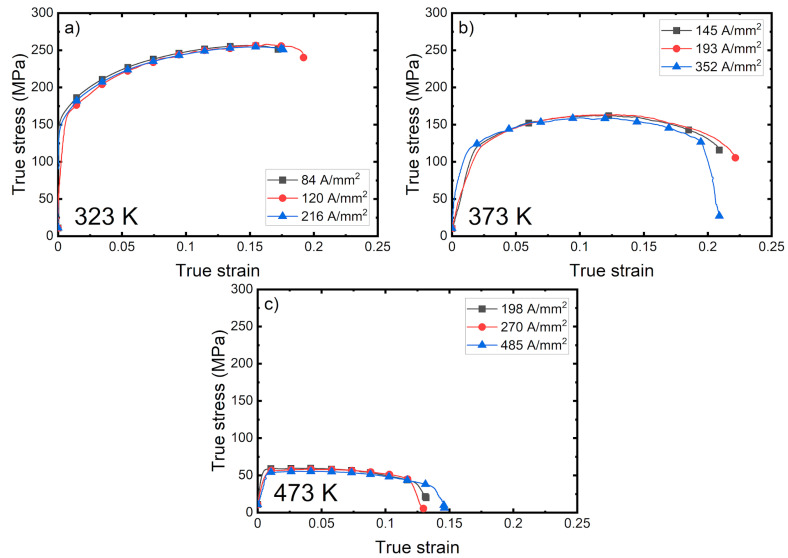
True stress–strain curve for (**a**) 323 K, (**b**) 373 K, and (**c**) 473 K.

True stress–strain curves can be seen in Figure 7a–c for 323 K, 373 K and 473 K, respectively. All the stress–strain curves show a similar shape at each temperature. It is not apparent that there are changes in the stress. This result suggests that the stress reduction seen with the electricity-assisted formation mechanism is mainly caused by temperature, rather than current density.

**Figure 8:**
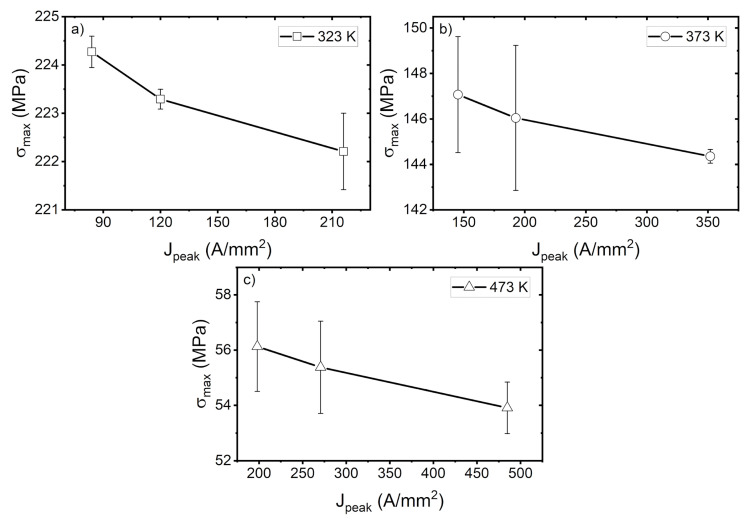
Ultimate tensile strength at various peak current densities at (**a**) 323 K, (**b**) 373 K, and (**c**) 473 K.

The ultimate tensile strength (UTS) for each parameter is shown in Figure 8. It shows that the UTS was falling as the peak current density increased. At 323 K, as can be seen in Figure 8a, the difference in UTS between the lowest and highest peak current density is 2.06 MPa. In Figure 8b, we see a similar trend in UTS, where the difference at 373 K is 2.72 MPa. Finally, for 473 K in Figure 8c, the trend persists, with a difference of 2.22 MPa. These results suggest that the reduction in UTS is independent of temperature. There is the possibility of achieving less stress with a higher peak current density regardless of the working temperature. This is beneficial in terms of avoiding unnecessary oxidation at the material’s surface, while still maintaining low stress.

The Joule heating effect was successfully minimized by maintaining a similar temperature at different peak current densities. This means Joule heating is not the only reason for the stress reduction. The other consideration is electron wind force causing electromigration [16]. This theory involves the transfer momentum of electrons moving towards dislocation.

Moving electrons in a conductor scatter when they collide with a dislocation, thus producing extra force in the movement of dislocation [16]. This effect can be stated by

(6) $$F_{ew}=\frac{\rho_{D}}{N_{D}}en_{e}J$$

where $F_{ew}$ is the force per unit length of dislocation, e is the electronic charge, J is the current density, and $n_{e}$ is the density of the free electrons.

The effect of electron wind was reported in studies on the springback reduction of a Ti-6Al-4V titanium alloy sheet [9]. The athermal effect can promote a dislocation motion and the unraveling of dislocation pileups. Specimens with a higher peak current density at the same RMS value displayed less dislocation entanglement.

However, the effect of electron wind is considered lower than Joule heating [17]. Using a numerical approach, Lahiri [18] showed that the effect of the electron wind force is negligible. Both Molotskii and Lahiri proposed the de-pinning of dislocation via the magnetic field that is generated while the current is flowing in the conductor [17,18,19]. However, both these studies were undertaken using face-centered cubic (fcc) metals; this study proves that the effect of an induced magnetic field on plasticity is also observable in hcp metals.

**Figure 9:**
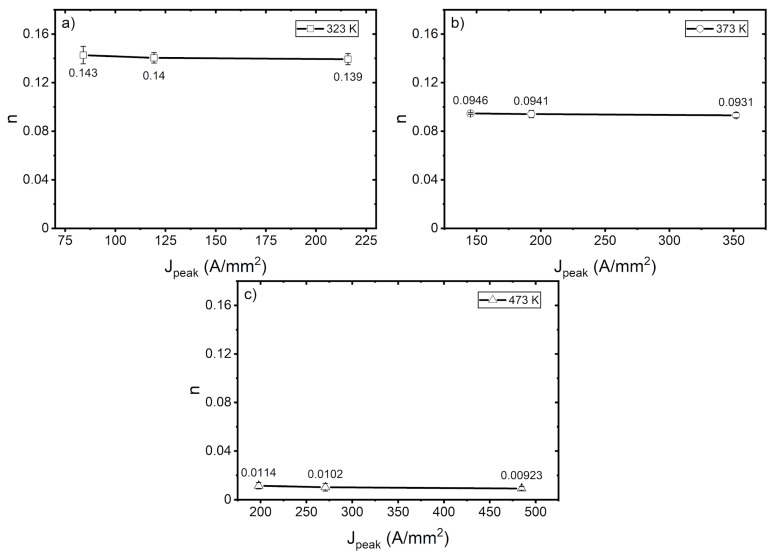
Strain hardening exponent at various peak current densities at (**a**) 323 K, (**b**) 373 K, and (**c**) 473 K.

The magnetic field-induced transition between the S and T states of the pinning bonds can significantly facilitate the depinning. This mechanism can prevent the dislocation from tangling into forest dislocation. The strain hardening exponent also exhibited a slight decreasing trend with increasing peak current density, as can be seen in Figure 9. This result supports previous observations of UTS. From the discussion, we can conclude that a small reduction in stress can be attributed to the dislocation density. Since this study uses normalized specimens, it can be assumed that the dislocation density is relatively low, so as to not form many forest dislocations.

**Figure 10:**
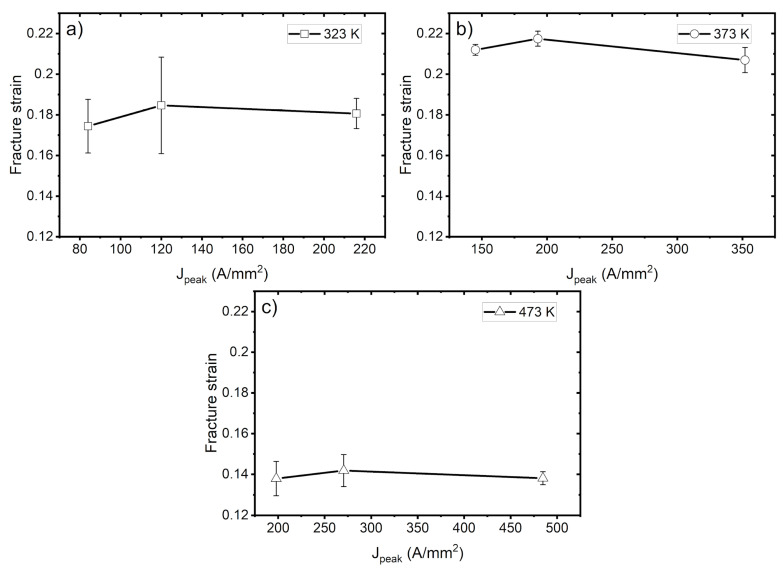
Fracture strain at various peak current densities at (**a**) 323 K, (**b**) 373 K, and (**c**) 473 K.

Figure 10 shows the fracture strain for different peak current densities. At a low peak current density, the fracture strain shows signs of improvement in terms of elongation. However, at a certain point, it begins to show a decreasing tendency. This behavior was observed at all temperatures, as shown in Figure 10a–c for 323 K, 373 K and 473 K, respectively. Bao et al. [20] found that when the electropulse reaches a certain value, there is a reduction in fracture strain. This reduction occurs at a peak current density of 344 A/mm [2] and an RMS value of 28.1 A/mm [2]. Ghiotti et al. [21] reported similar results as regards fracture strain for a AA1050 aluminum alloy. After being subjected to a DC flow of 5 A/mm [2] current density, the cooled specimens showed a reduction in fracture strain. The same tendencies were also observed in other rolling directions. It is suggested that to improve elongation, an optimum value of pulsed current exists.

At a high current density, especially with a pulsed current, a nonhomogeneous distribution of current density can occur. This nonhomogeneous distribution can be called the current crowding effect (CCE). The formation of voids was accelerated in regions with higher current densities compared to others. This non-uniform distribution of current density results in a non-uniform local temperature distribution, resulting in the retardation of elongation. Hieu and Salm [22] explained that the CCE will have a greater effect when the dimensions are reduced. As such, the CCE will have a greater effect under high strain conditions when the specimen’s dimensions are reduced. The authors also argued that it is important to avoid current crowding so as to avoid early failures. [22,23] From these considerations, it is inferred that at high enough current densities, the CCE can become significant, causing specimens to fail earlier.

## 4. Conclusions

This work presents the influence of pulsed current on the tensile properties of an AZ31B magnesium alloy. It was observed the value of the ultimate tensile strength decreased with the increase in peak current density, regardless of its temperature. This proves the athermal effects of the pulsed current, which affect tensile properties. The strain hardening exponent also displayed a reducing tendency at higher current densities. For fracture strain, it seems there is an optimum pulsed current condition for improving its elongation. This study has demonstrated the existence of the athermal effect of pulsed current.

## Figures and Tables

**Table 1:** Chemical composition (wt.%) of AZ31B magnesium alloy.

Al	Zn	Mn	Si	Fe	Cu	Ni	Ca	Mg
3.1	0.88	0.37	0.01	0.002	<0.01	<0.001	0.01	Rem.

**Table 2:** Experimental parameters.

Target Temperature	Pulse Width	Strain Rate	Frequency
(K)	(µs)	(1/s)	(Hz)
323	150	0.001	200
			600
			1000
373			200
			600
			1000
473			200
			600
			1000

## Data Availability

The data presented in this study are available on request from the corresponding author. The data are not publicly available as the data also forms part of an ongoing study.
